# Nosocomial SARS-CoV-2 Infections in Japan: A Cross-sectional Newspaper Database Survey

**DOI:** 10.34172/ijhpm.2020.75

**Published:** 2020-05-20

**Authors:** Yuta Tani, Toyoaki Sawano, Ayumu Kawamoto, Akihiko Ozaki, Tetsuya Tanimoto

**Affiliations:** ^1^Medical Governance Research Institute, Tokyo, Japan.; ^2^Department of Surgery, Sendai City Medical Center, Sendai, Japan.; ^3^Faculty of Medicine, University of Szeged, Szeged, Hungary.; ^4^Department of Breast Surgery, Jyoban Hospital of Tokiwa Foundation, Fukushima, Japan.

## Dear Editor,

An outbreak of coronavirus disease in 2019 (COVID-19) caused by the severe acute respiratory syndrome coronavirus 2 (SARS-CoV-2) has become a pandemic.^1^ In Japan, the increasing number of patients infected with SARS-CoV-2 threatens pre-existing medical resources, and incidents of group nosocomial infections in medical institutions and infected healthcare professionals (HCPs) are also increasing. Using the largest domestic newspaper database, we summarized the number of reported nosocomial infections of SARS-CoV-2 occurring throughout Japan.

As done elsewhere,^2^ we collected articles published from February 15, 2020 to April 6, 2020, from four nationwide newspapers, two major news agencies, and 53 local newspapers by searching the largest newspaper database in Japan, Nikkei Telecom,^3^ using the Japanese keywords “*shin- gata -korona- uirusu*” (novel coronavirus) and “*in- nai - kansen*” (nosocomial infection) and one of the following three Japanese keywords: “*ishi*” (physician), “*kan -ja*” (patient), and “*kango-shi*” (nurse). Next, we selected articles based on the titles including certain keywords, namely “*in- nai - kansen*” (nosocomial infection’) or “*byo -in*” (hospital), and numbers of infected cases being disclosed, as described in Supplementary file 1. The overall number of cases among the public living in Japan was retrieved from the Ministry of Health, Labour and Welfare.^4^

In the initial screening process, we identified 1216 articles, and two authors eventually selected 79 articles (6.5%) for the study. The total number of nosocomial infections was 246 in 17 medical institutions in 10 prefectures (Table 1), accounting for 6.9% of the total number of cases testing positive in Japan as of April 6, 2020 (n = 3569), excluding those of the Diamond Princess cruise ship.^5^

**Table 1:** Reported Cases of SARS-CoV-2 Nosocomial Infections in Japan

**Medical Institutions** **(Number of General/Infectious Disease Beds)**	**Prefecture**	**Number of Nosocomial Infections**				
		**Physicians** **(n = 63)**	**Patients** **(n = 46)**	**Office Workers** **(n = 5)**	**Unidentified** **(n = 132)**	**Total** **(n = 246)**
Eiju Hospital (405/0)	Tokyo	Not available	Not available	Not available	128 (100.0%)	128 (52.0%)
Oita Medical Center (300/0)	Oita	9 (37.5%)	9 (37.5%)	2 (8.3%)	4 (16.7%)	24 (9.8%)
Shin Komonji Hospital (214/0)	Fukuoka	19 (95.0%)	1 (5.0%)	0 (0.0%)	0 (0.0%)	20 (8.1%)
Ja Toride Medical Centre (408/8)	Ibaraki	3 (25.0%)	9 (75.0%)	0 (0.0%)	0 (0.0%)	12 (4.9%)
Fukuoka Kinen Hosipital (239/0)	Fukuoka	10 (83.3%)	2 (16.7%)	0 (0.0%)	0 (0.0%)	12 (4.9%)
Jinkei Hospital (211/0)	Hyogo	3 (27.3%)	8 (72.7%)	0 (0.0%)	0 (0.0%)	11 (4.5%)
Keio University Hospital (960/0)	Tokyo	2 (28.6%)	5 (71.4%)	0 (0.0%)	0 (0.0%)	7 (2.9%)
Mashimo Cinic (0/0)	Gunma	2 (33.3%)	1 (16.7%)	3 (50.0%)	0 (0.0%)	6 (2.4%)
The Jikei University Hospital (1026/0)	Tokyo	4 (66.7%)	2 (33.3%)	0 (0.0%)	0 (0.0%)	6 (2.4%)
Takarazuka Daiichi Hospital (199/0)	Hyogo	2 (40.0%)	3 (60.0%)	0 (0.0%)	0 (0.0%)	5 (2.0%)
Saiseikai Arita Hospital (184/0)	Wakayama	2 (50.0%)	2 (50.0%)	0 (0.0%)	0 (0.0%)	4 (1.6%)
Tatebayashi Kosei General Hospital (323/6)	Gunma	3 (100.0%)	0 (0.0%)	0 (0.0%)	0 (0.0%)	3 (1.2%)
Fukuchiyama Shimin Hospital (344/4)	Kyoto	2 (66.7%)	1 (33.3%)	0 (0.0%)	0 (0.0%)	3 (1.2%)
Sagami Chuo Hospital (16/00)	Kanagawa	0 (0.0%)	2 (100.0%)	0 (0.0%)	0 (0.0%)	2 (0.8%)
Oita Prefectural Hospital (566/12)	Oita	1 (100.0%)	0 (0.0%)	0 (0.0%)	0 (0.0%)	1 (0.4%)
Chubu Rosai Hospital (556/0)	Aichi	0 (0.0%)	1 (100.0%)	0 (0.0%)	0 (0.0%)	1 (0.4%)
Yokosuka City Hospital (476/6)	Kanagawa	1 (100.0%)	0 (0.0%)	0 (0.0%)	0 (0.0%)	1 (0.4%)

Abbreviation: SARS-CoV-2, severe acute respiratory syndrome coronavirus.

The number of infected cases in each hospital varied from 1 to 128, and the maximum number (52.0%) was found in the Eiju Hospital in Tokyo. This means that nosocomial infections amounted to 141 (13.6%) among the 1040 infected cases in Tokyo. Of the 114 cases with known details of infection, 63 (55.3%) were HCPs, 46 (40.4%) were patients, and five (4.4%) were office workers and their families.Details of the remaining 132 cases have not been identified.

Details of nosocomial infections in the four hospitals were extracted from the newspaper articles at the authors’ discretion as representative cases of nosocomial infection in Japan (Table 2). The causes of nosocomial infection were largely categorized into insufficient patient isolation and HCP protection. A typical example of insufficient patient isolation occurred at the JA Toride Medical Center, wherein an undiagnosed patient infected another patient in the same room. The initial patient was diagnosed with pneumonia, but reverse transcription polymerase chain reaction (RT-PCR) testing for SARS-CoV-2 was not performed upon admission due to the limited capacity of this testing.^6^ Similarly, a typical example of insufficient HCP protection occurred at Shin Komonji Hospital, wherein a patient who received emergency surgery before being diagnosed with COVID-19 subsequently infected 16 HCPs who worked with simple personal protective equipment (eg, masks) in the ward.

**Table 2:** Representative Causes for Nosocomial Infections and Conceivable Countermeasures^6^

**Representative Causes**	**Medical Institutions**	**Details of Nosocomial Infections**	**Conceivable Countermeasures**
Insufficient patient isolation	Keio University hospital	An undiagnosed asymptomatic patient transferred from Eiju hospital infected three other patients in the same room in the ward. RT-PCR testing was not offered to the patient when transferred.	- RT-PCR should be offered to all patients transferred from other hospitals with known SARS-CoV-2 cases. - Rigorous RT-PCR testing should be performed before admission in an endemic area. - Interventions for patients without a negative test result for SARS-CoV-2 should be performed in a negative-pressure or well-ventilated ward.
	JA Toride Medical Centre	A patient was diagnosed with pneumonia upon admission, but RT-PCR was not offered because of the limited capacity of RT-PCR testing.	
Insufficient HCP protection	Saiseikai Arita Hospital	An attending physician was infected by a surgical ward patient who was later found to be infected with SARS-CoV-2.	- Increase awareness of personal protection, sufficient personal protective equipment, and proper preparedness and response among HCPs. - HCPs should highlight primary protection, such as standard and droplet precautions, whenever in the work environment. - HCPs should undertake secondary protection, namely any type of prevention relating to contact with patients, especially when performing invasive procedures.
	Shin Komonji Hospital	Sixteen HCPs who worked with simple personal protective equipment (eg, surgical masks) were infected by a patient who received emergency surgery and was subsequently diagnosed with SARS-CoV-2.	

Abbreviations: RT-PCR, reverse transcription polymerase chain reaction; SARS-CoV-2, severe acute respiratory syndrome coronavirus; ICU, intensive care unit; HCPs, healthcare professionals.

At least 6.9% of the total number of SARS-CoV-2 cases throughout Japan were reported in newspapers as nosocomial infections, and most occurred in relatively large hospitals. Nosocomial infections of SARS-CoV-2 have been reported in other countries in part as a result of a lack of equipment and poor medical practices,^7,8^ and in China, these infections have amounted to 3.8% of all cases.^7^ However, Japan’s hospital environment may be prone to nosocomial infections because physicians care for many patients and often work at multiple clinics and hospitals due to the relatively small number of physicians in the country. Therefore, if infected, physicians themselves might convey the virus to multiple medical institutions. Furthermore, hospitals usually treat multiple patients in crammed rooms in their wards. In some cases, an initially hospitalized patient who did not have a confirmatory diagnosis at the time of admission subsequently spread the virus to other patients in the same room as well as to HCPs. In Eiju Hospital, 163 infected cases and 20 deaths due to COVID-19 have been reported as of April 11, 2020.^9^

Countermeasures against nosocomial infections should be considered based on their primary causes. First, with regard to insufficient patient isolation, a negative pressure ward should be available for patients with suspected, but not confirmed, SARS-CoV-2, and when this is unavailable, frequent ventilation should be encouraged in such a ward as much as possible.^6^ Second, with regard to insufficient HCP protection, thorough primary protection, such as standard and droplet precautions, should be employed.^10^ When performing invasive procedures for COVID-19 patients, HCPs should undertake secondary protection, namely infection control rules relating to contact with patients, such as wearing gloves, hand hygiene, and wearing a gown.^6,10^

Historically, Middle East respiratory syndrome coronavirus (MERS-CoV) infection has caused outbreaks, including through nosocomial infections, in countries such as South Korea.^11^ It is crucial to be aware of the risks associated with infected patients because coronavirus infections, including both SARS-CoV-2 and MERS-CoV, spread via human-to-human transmission, and SARS-CoV-2 is thought to have a higher basic reproduction number than MERS-CoV (2.24 to 3.58 vs. 0.52 to 1.36).^12,13^ Thus, HCPs should follow rigorous practices to deal with SARS-CoV-2; selection, isolation, protection, and observation of medical personnel with appropriate examination routines.^14^ In addition, they should observe diagnosis criteria and carry out examination routines for suspected infected patients.^14^

Several limitations are present in our study. First, reporting in newspapers can be inaccurate. Second, definitions of nosocomial infections may differ among newspaper companies and articles. Third, our study may have not covered all nosocomial infections due to underreporting.

In conclusion, nosocomial infections of SARS-CoV-2 are common in Japan and have spread among several medical institutionsvia infected patients and HCPs. HCPs need to develop flexible strategies and action plans to deal with the current pandemic and prevent its further spread.

## Ethical issues

Not applicable.

## Competing interests

Drs. Ozaki and Tanimoto report personal fees from MNES Inc. All other authors declare that they have no competing interests.

## Authors’ contributions

YT, TS, AK, AO, and TT wrote the manuscript. YT and AO carried out data collection and screening. Each author has participated sufficiently in the work and given final approval of the submitted manuscript. All authors have made substantial contribution to the content of the manuscript in various sections.

## Authors’ affiliations

^1^Medical Governance Research Institute, Tokyo, Japan. ^2^Department of Surgery, Sendai City Medical Center, Sendai, Japan. ^3^Faculty of Medicine, University of Szeged, Szeged, Hungary. ^4^Department of Breast Surgery, Jyoban Hospital of Tokiwa Foundation, Fukushima, Japan.

## Supplementary files


Supplementary file 1. Details of the Algorithm Used for Screening Articles.
