# Supplementary material for: Nosocomial SARS-CoV-2 Infections in Japan: A Cross-sectional Newspaper Database Survey
Source: Int J Health Policy Manag. 2020 May 20;9(10):461–3. doi: 10.34172/ijhpm.2020.75 (PMC7719222; doi:10.34172/ijhpm.2020.75)
Supplement: Supplementary file 1 — Details of the Algorithm Used for Screening Articles. [file ijhpm-9-461-s001.pdf]

Supplementary file 1. Details of the Algorithm Used for Screening Articles

We collected articles published between February 15, 2020 and April 6, 2020 in four nationwide newspapers, two major news agencies, and 53 local newspapers by searching the largest newspaper database in Japan, Nikkei Telecom. To search for the articles, we used the Japanese keywords "*shin-gata-korona-uirusu*" (novel coronavirus) and "*in-nai-kansen*" (nosocomial infection) and one of the following three Japanese keywords: "*ishi*" (physician), "*kan-ja*" (patient), or "*kango-shi*" (nurse). Using this process, 1216 articles were retrieved for further assessment. Of these articles, we selected those with the words "*in-nai-kansen*" (nosocomial infection) or "*byo-in*" (hospital) in the headline and reporting on the number of COVID-19 cases in such hospitals. At this point, 126 of the 1216 articles were extracted. Thereafter, duplicates were removed as follows: if the articles were published on the same day and had the same hospital or region names, we regarded them as duplicates. In cases of duplications, we prioritized the following articles: 1. Articles published by the four major newspapers or two major news agencies, and 2. Articles having a greater number of keywords in the headline. We included the most recently published articles if the duplications published differing numbers of cases. Finally, we extracted 79 articles (6.5%) out of the 1216 articles originally retrieved. Medical institutions with only one case were also judged to be a nosocomial infection as long as the patients were reported to have nosocomial infections.
